# ChatGPT versus expert psychiatrist in managing mixed mania: a comparative case study

**DOI:** 10.1192/j.eurpsy.2026.11180

**Published:** 2026-07-31

**Authors:** F. Zaouali, I. Anes

**Affiliations:** 1 Emergency department and mobile emergency and resuscitation service, Tahar Sfar University Hospital, Mahdia; 2 Outpatient psychiatric consultation, Hadj Ali Soua Regional Hospital, Ksar Hellal, Monastir, Tunisia

## Abstract

**Introduction:**

Artificial intelligence (AI) models, such as ChatGPT, are increasingly explored for clinical decision support in psychiatry. Mixed mania, featuring simultaneous manic and depressive symptoms, poses diagnostic and therapeutic challenges. The reliability of AI recommendations for such complex cases remains largely unknown.

**Objectives:**

To evaluate ChatGPT’s performance in managing mixed mania by comparing its responses to two clinical cases with those of a senior psychiatrist, assessing adherence to current clinical guidelines.

**Methods:**

Two anonymized mixed mania case vignettes were presented to ChatGPT (GPT-4.0) and a senior psychiatrist. Responses were evaluated independently by two psychiatrists for: diagnostic accuracy, appropriateness of pharmacological and non-pharmacological recommendations, and guideline adherence. Discrepancies were resolved by consensus. Performance metrics included agreement with guidelines and concordance between ChatGPT and the human expert.

**Results:**

ChatGPT correctly identified the diagnosis in 1 of 2 cases; the senior psychiatrist achieved 2/2 correct diagnoses. Pharmacological recommendations by ChatGPT partially aligned with guidelines, suggesting first-line mood stabilizers but insufficiently addressing comorbid depressive symptoms. Non-pharmacological advice was general and lacked individualized psychosocial interventions. Overall guideline adherence was 65% for ChatGPT versus 95% for the psychiatrist. Concordance between ChatGPT and the psychiatrist was moderate (Cohen’s kappa = 0.56).

**Conclusions:**

ChatGPT shows partial capability in recognizing and managing mixed mania, with moderate guideline adherence. AI may offer preliminary guidance, but expert psychiatric evaluation remains essential for complex mood disorders. Further AI refinement and training on nuanced psychiatric cases are needed to improve reliability and clinical utility.

**Disclosure of Interest:**

None Declared

